# High-resolution phase-contrast imaging of biological specimens using a stable betatron X-ray source in the multiple-exposure mode

**DOI:** 10.1038/s41598-019-42834-2

**Published:** 2019-05-24

**Authors:** Bo Guo, Xiaohui Zhang, Jie Zhang, Jianfei Hua, Chih-Hao Pai, Chaojie Zhang, Hsu-Hsin Chu, Warren Mori, Chan Joshi, Jyhpyng Wang, Wei Lu

**Affiliations:** 1 Tsinghua University, Department of Engineering Physics, Beijing, 100084 China; 2 University of California Los Angeles, Department of Electrical Engineering, Los Angeles, California 90095 USA; 3 National Central University, Department of Physics, Taoyuan, 32001 Taiwan; 4 University of California Los Angeles, Department of Physics and Astronomy, Los Angeles, California 90095 USA; 5 Institute of Atomic and Molecular Sciences, Academia Sinica, Taipei, 10617 Taiwan; 6 National Taiwan University, Department of Physics, Taipei, 10617 Taiwan

**Keywords:** Plasma-based accelerators, X-rays

## Abstract

Phase-contrast imaging using X-ray sources with high spatial coherence is an emerging tool in biology and material science. Much of this research is being done using large synchrotron facilities or relatively low-flux microfocus X-ray tubes. An alternative high-flux, ultra-short and high-spatial-coherence table-top X-ray source based on betatron motions of electrons in laser wakefield accelerators has the promise to produce high quality images. In previous phase-contrast imaging studies with betatron sources, single-exposure images with a spatial resolution of 6–70 *μ*m were reported by using large-scale laser systems (60–200 TW). Furthermore, images obtained with multiple exposures tended to have a reduced contrast and resolution due to the shot-to-shot fluctuations. In this article, we demonstrate that a highly stable multiple-exposure betatron source, with an effective average source size of 5 *μ*m, photon number and pointing jitters of <5% and spectral fluctuation of <10%, can be obtained by utilizing ionization injection in pure nitrogen plasma using a 30–40 TW laser. Using this source, high quality phase-contrast images of biological specimens with a 5-*μ*m resolution are obtained for the first time. This work shows a way for the application of high resolution phase-contrast imaging with stable betatron sources using modest power, high repetition-rate lasers.

## Introduction

Since the discovery of X-rays by Röntgen in 1896, X-ray based imaging technologies have been indispensible in modern medical, biological and material research. The standard imaging techniques based on X-ray absorption (the imaginary part of the refractive index, *n* = 1 − *δ* + *iβ*), such as radiography and computed tomography (CT), rely on the variations of X-ray absorption in matter to obtain a high contrast, thereby limiting their usefulness to samples/structures that have high absorption gradients^1,2^. On the other hand, imaging techniques based on X-ray phase variations (the real part of the refractive index), such as phase-contrast imaging (PCI), can produce high contrast images of low atomic number samples that have little variation in absorption, such as many soft tissues of biological importance^3,4^. For this reason, the PCI technique, especially the in-line PCI scheme, has attracted significant attention in the past two decades in biological and material imaging^5–7^. One key requirement for making high quality in-line PCI images is good spatial coherence of the X-ray source^8,9^. In practice, only X-ray tubes with a micro focus or large synchrotron facilities can provide a sufficient level of spatial coherence to be useful. However, both technologies have their own limitations: the former has very low photon flux and thus needs long exposure times, and access to the latter is limited by high user demand on these large facilities and high cost^10^. To make in-line PCI more convenient and affordable, a compact high-flux X-ray source with high spatial coherence is therefore needed^11^. Fortunately, a new type of directional X-ray source belonging to this category called betatron radiation is being explored and showing considerable promise for this purpose^12–14^. Here the X-rays are generated in the synchrotron-like radiation emitted by relativistic electrons in a compact laser wakefield accelerator (LWFA).

Betatron radiation is simply a by-product of a laser or beam driven wakefield accelerator^12,15,16^. In a LWFA, an ultra-short intense laser pulse propagating in a low density plasma creates a strong wake by pushing the plasma electrons away from its path, forming an ion cavity-like structure^17^. This wake carries extremely strong accelerating and focusing fields that can approach 100 GV/m level, which is nearly three orders of magnitude larger than those of conventional accelerators. Electrons can be injected into this wake and gain hundreds of MeV energy in just a few millimeters^18–20^, enabling a compact source of relativistic electrons from a tabletop-scale device. These high energy electrons also oscillate transversely (called betatron oscillation) under the immense focusing fields of the wake, very similar to the transverse electron motion in a synchrotron wiggler, thereby creating a bright synchrotron-like X-ray radiation (called betatron radiation) in the near forward direction within a small angle of tens of milliradians^14^. Since the typical betatron oscillations have *μ*m-level amplitudes, the betatron sources can have effective source sizes of the same order^13,21,22^, making high quality imaging with *μ*m-level resolution possible.

In the past decade, the development of the betatron source based on LWFA has witnessed rapid progress, with both the high peak brightness (>10^22^ photons/s/mrad^2^/mm^2^/0.1%BW, comparable with the third-generation synchrotron sources) and the *μ*m-level source size confirmed experimentally^13^. Furthermore, preliminary phase-contrast imaging experiments using betatron radiation from self-injection LWFA have been carried out on insect^23–25^ and mouse^26^ samples. In these experiments, modest single-exposure image resolutions of 6–70 *μ*m were obtained by using relatively large lasers (60–200 TW). It is also reported that when images were obtained with multiple exposures the spatial resolution and the contrast degraded greatly due to the large shot-to-shot fluctuations of the source parameters in self-injection LWFA^23^. For practical application of imaging, stable betatron sources using lower power lasers (<40 TW) are highly preferred. Such lasers, based on the Ti:sapphire medium, are typically more affordable and compact compared to lasers with >100-TW power and therefore exist in numerous laboratories. In this paper, we show that a highly stable betatron source powered by a relatively modest power (30–40 TW) Ti:sapphire laser, with a photon number and pointing jitter better than 5% and spectral fluctuation less than 10%, can be obtained by combining ionization injection^27–29^ and multiple-exposure mode. Furthermore, due to the much smaller position fluctuation of the X-ray source in ionization injection compared with self-injection, multiple-exposure phase-contrast images of biological specimens with 5-*μ*m resolution have been obtained for the first time.

## Results

### Experimental setup and basic measurement

**Figure 1:**
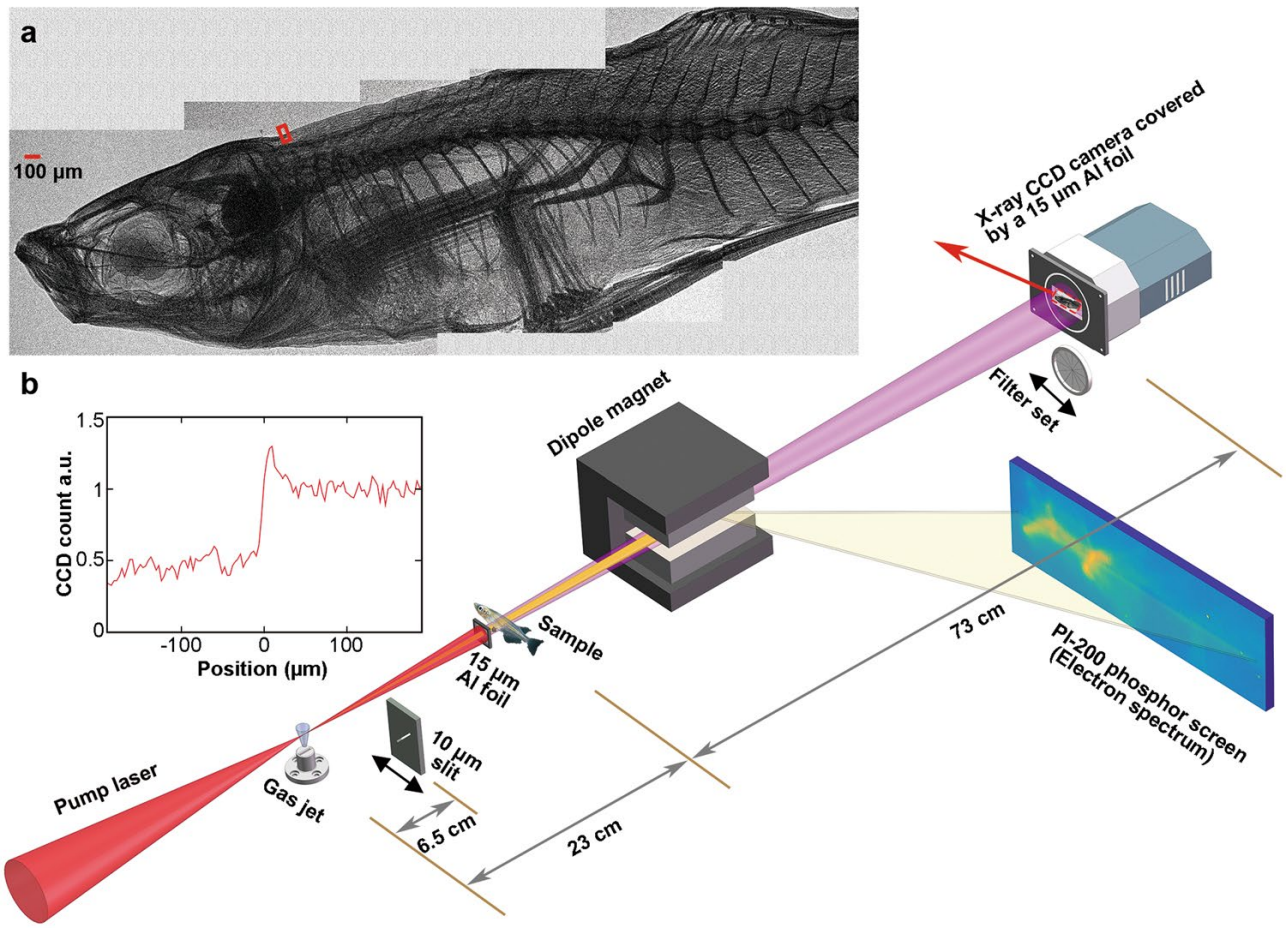
Schematic of the in-line phase-contrast imaging experimental layout. (**a**) A phase-contrast image of a small fish (1-cm long). The whole image was generated by 5 sub-images taken separately, and each sub-image was obtained by accumulation of 20 exposures. (**b**) Edge lineout for the back-fin marked by small red box in (**a**) showing a clear edge enhancement characteristic of PCI.

The experimental layout is shown in Fig. 1. The experiment was carried out with the ultra-short high power Ti:sapphire laser system at National Central University^30^. The 30–40-TW driving laser pulse (energy 1.1–1.4 J, FWHM duration 36 fs) was focused using an f/8.3 off-axis parabolic mirror, with 50% energy enclosed in a Gaussian fitted spot of ∼16-*µ*m (FWHM) diameter. The target used for producing the plasma was a supersonic nitrogen (for ionization injection) or helium (for self-injection) rectangular gas jet with a dimension of 4 × 0.15 mm^2^, and the plasma electron density was measured online by a Mach-Zehnder interferometer using a probe laser pulse. During the experiment, relativistic electron beams generated in the laser wakefield accelerator were deflected by a 0.8-Tesla 8-cm long permanent dipole magnet onto a phosphor screen for the measurement of the energy spectra. The betatron X-rays were recorded by a back-illuminated X-ray CCD camera (ANDOR DX434-BN) mounted 96 cm away from the gas jet in vacuum and covered by a 15-*µ*m aluminum foil to block the residual laser light. The betatron X-ray profile and photon number can be directly measured with this camera.

For the measurement of X-ray spectrum, a filter set consisting of 20 Al foils with different thickness (6–80 *µ*m) was inserted in the X-ray path to record the attenuation profile on the X-ray CCD, which then can be used to deduce the X-ray spectrum using the expectation maximization (EM) algorithm^31^. By taking into account the transmission of the Al foils and the quantum efficiency of the X-ray CCD, X-rays within an energy range 4 keV to 15 keV can be measured. To measure the position fluctuation of betatron source, a 10-*µ*m slit with an angle of 45° relative to the horizontal direction was placed 6.5 cm away from the gas jet along the X-ray path and imaged by the X-ray CCD, yielding a 13.8-fold magnification of the position fluctuation of betatron source at the CCD plane. The position fluctuation was measured in the direction perpendicular to the slit. For the measurement of phase-contrast images, small specimens covered by a 15-*µ*m Al foil was placed 23 cm away from the gas jet in vacuum along the laser path, with a sample-detector distance of 73 cm (the calculation of optimal source-sample and sample-detector distance is expressed in Methods). High quality phase-contrast images of a small fish and a butterfly have been obtained in this experiment in multiple-shot exposure mode. In Fig. 1a, a PCI image of a small fish (1-cm long) is shown, where very minute features like small bones and skins are clearly visible with high contrast. We note that due to the limited field of view of the X-ray CCD (14 × 14 mrad^2^), the whole image is generated by 5 sub-images taken separately, and each sub-image was obtained by accumulation of 20-shot exposure. A lineout of a small portion of the image within the red box is shown in Fig. 1b, where the clear edge enhancement characteristic of PCI is evident.

### Betatron X-ray generation based on ionization injection

To obtain a stable betatron source, a systematic optimization has been performed by adjusting the laser pulse duration, the focal position and the plasma density. It was found that an optimal betatron X-ray can be produced at a plasma electron density near 1.0 × 10^19^ cm^−3^ (nitrogen atom density *n*_*N*_ = 2.0 × 10^18^ cm^−3^) with relatively low energy electron beams (up to 70 MeV) when the laser pulse with an energy of 1.1–1.4 J was focused near the front edge of the gas jet. Run at a lower plasma density (5.0 × 10^18^ cm^−3^), however, electron beams with higher energy (up to 250 MeV) can be produced but with weak betatron radiation.

**Figure 2:**
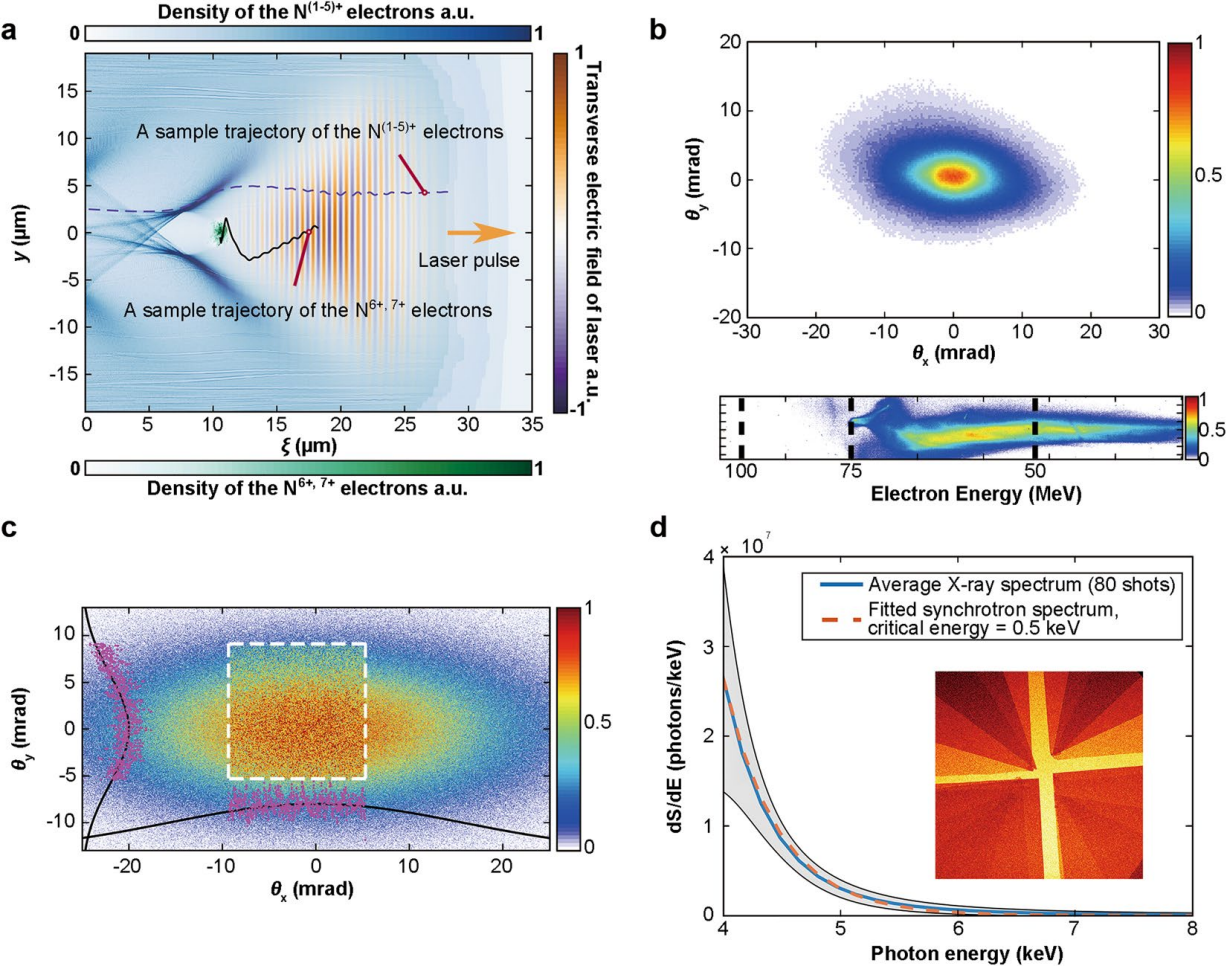
(**a**) 2D particle-in-cell simulation of ionization injection with OSIRIS using experiment parameters (nitrogen atom density *n*_*N*_ = 2.0 × 10^18^ cm^−3^, normalized laser vector potential *a*_0_ = 2.17). Sample trajectories of the outer N^(1–5)+^ and the inner N^6+,7+^ electrons are shown. (**b**) The experimentally observed elliptical electron profile and the electron energy spectrum with a broad energy spread. (**c**) A typical single-exposure betatron X-ray profile also with an elliptical shape. The portion within the white-dotted square represents the field of view of the X-ray CCD. The overall elliptical distribution can be fitted with a bi-Gaussian function. (**d**) A measured average X-ray spectrum (blue solid line) with the error bars (standard deviation, grey region) of 80 consecutive shots and its fitted synchrotron spectrum (orange dashed line) with a critical energy of 0.5 keV. The inset is the actual X-ray profile measured with the filter set, which can be used to deduce the X-ray spectrum.

To clearly understand the physical process of ionization injection in the experiment, two dimensional particle-in-cell simulations using the experimental parameters have been performed with the code OSIRIS^32^ that implements the ADK theory^33^ to model the ionization process. In Fig. 2a, the laser electric field strength and the electron density generated by ionization from one sample simulation are shown to illustrate the ionization injection process. The first five electrons of nitrogen atom are easily ionized by the leading edge of the laser to form the background plasma with an electron density of 1.0 × 10^19^ cm^−3^. Due to their much higher ionization potential, the 6^th^ and 7^th^ nitrogen electrons can only be ionized inside the wake where the laser intensity is near its peak. These electrons then keep gaining energy from the wakefield and can get trapped in the wake during their slipping back towards the tail of the bubble. Since the ionization and acceleration processes occur continuously, the injected electron beams typically have a broad energy spectrum with a smooth and continuous shape, which can be observed directly from the experimentally measured spectrum (Fig. 2b). Figure 2b also shows a typically measured electron beam profile of an elliptical shape, with the major axis along the laser polarization direction. This asymmetry is mainly caused by the direct interaction of the injected electrons with the driving laser, which also leads to a similar elliptical shape for the resultant betatron X-ray profile^29,34^. A typical single-exposure X-ray profile with an elliptical shape^35^ is shown in Fig. 2c, where the portion within the white-dotted square represents the field of view of the X-ray CCD. The centroid position and the divergence of the X-ray beam can be obtained from the whole fitted profile. On Fig. 2d, we also plot a measured X-ray spectrum (80 consecutive shots average) and its fitted synchrotron spectrum with a critical energy of 0.5 keV. Combining the fitted synchrotron spectrum with the total deposited X-ray energy on the CCD, an average total photon number of 1.1 × 10^7^ photons/shot (4–15 keV) has been measured, which also gives an estimation of 2.2 × 10^8^ photons/shot for the energy range 1–15 keV.

### Improved stability by combining ionization injection with multiple exposures

**Figure 3:**
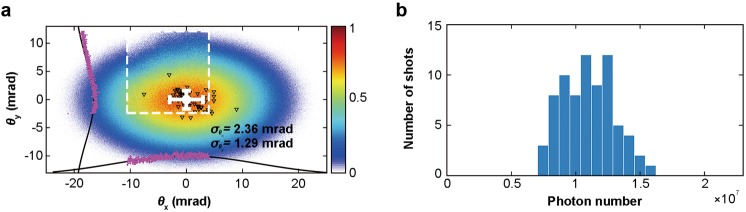
Statistics of X-ray characteristics of 80 consecutive shots. (**a**) Average angular X-ray profile and each centroid position (black triangles) of the 80 consecutive shots showing a pointing jitter less than 10% of the average divergence (25.2 × 17.2 mrad^2^). (**b**) Photon number (4–15 keV) statistics of the same 80 shots as in Fig. 2d. The standard deviation is 1.8 × 10^6^ photons/shot, corresponding to 16% of the average photon number.

To show the high stability of pointing and photon number in an ionization injection based betatron source, in Fig. 3a we plot the centroid positions of 80 consecutive shots under the same experimental conditions on top of their average X-ray profile, and in Fig. 3b we plot the statistics of photon number of the same 80 shots as Fig. 2d. The average profile in Fig. 3a has an elliptical shape with a vertical divergence of 17.2 mrad (FWHM) and a horizontal divergence of 25.2 mrad (FWHM) respectively. The angular fluctuations of the centroid positions have standard deviations of 1.29 mrad vertically (σ_*θy*_) and 2.36 mrad horizontally (σ_*θx*_), less than 10% of the overall divergences. In Fig. 3b, the photon number has a standard deviation of 1.8 × 10^6^ photons/shot, corresponding to 16% of the average photon number of 1.1 × 10^7^ photons/shot (4–15 keV). These levels of stability are as good as the best reported results using a gas mixture target^29^.

To further optimize the betatron sources based on stable ionization injection, we systematically studied the effect of exposure number on the pointing, photon number, spectrum and imaging quality. It turns out that the stability of the multiple-exposure betatron sources indeed can be significantly enhanced without losing its high resolution capability for phase-contrast imaging in the single-exposure mode.

**Figure 4:**
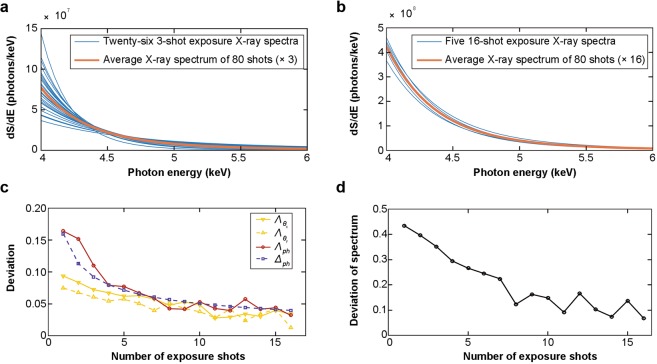
Stabilities of the *i*-exposure betatron X-ray sources. (**a**) 26 3-exposure spectra (blue solid lines) and their average spectrum (orange solid line). (**b**) 5 16-exposure spectra and their average spectrum (orange solid line). (**c**) Experimentally measured ${{\rm{\Lambda }}}_{{\theta }_{x}}$ (yellow solid line), ${{\rm{\Lambda }}}_{{\theta }_{y}}$ (yellow dashed line) and Λ_*ph*_ (red solid line) of *i*-exposure X-rays, and estimated Δ_*ph*_ (purple dashed line) of *i*-exposure X-rays. (**d**) Experimentally measured Λ_*S*_ of *i*-exposure X-rays.

To quantify the effect of exposure number, we have studied the trend of improvement of the multiple-exposure betatron X-ray sources based on a dataset of 80 consecutive shots (the same as Fig. 2d) under the same experimental condition. For a given number of exposures *i*, the 80 shots are divided into *n* groups, where *n* is the largest integer smaller than 80/*i*. For example, *i* = 3, *n* = 26 for the 3-exposure mode, and *i* = 16, *n* = 5 for the 16-exposure mode. To give a comparison, on Fig. 4a,b we plot the 26 3-exposure spectra and the 5 16-exposure spectra on top of the average spectrum of all the 80 shots (multiplied by 3 and 16) respectively. It is evident that the 16-exposure spectra (Fig. 4b) have much reduced deviation compared with the 3-exposure spectra (Fig. 4a).

For further analysis, the *i*-exposure photon number deviation can be defined as ${{\rm{\Lambda }}}_{ph}(i)={\sigma }_{{N}_{ph}}(i)/(i\times {\bar{N}}_{ph})$, where ${\sigma }_{{N}_{ph}}$(*i*) is the standard deviation of *n* groups of the *i*-exposure photon number, and ${\bar{N}}_{ph}$ is the average photon number of all the 80 shots. We can also define the *i*-exposure pointing deviation as ${{\rm{\Lambda }}}_{{\theta }}(i)={\sigma }_{{\theta }}(i)/\bar{{\theta }}$, where σ_*θ*_(*i*) is the standard deviation of *n* groups of the *i*-exposure centroid angular fluctuations (σ_*θx*_(*i*) for horizontal direction, σ_*θy*_(*i*) for vertical direction), and $\bar{{\theta }}$ is the average divergence of all the 80 shots (${\bar{{\theta }}}_{x}$ for horizontal direction, ${\bar{{\theta }}}_{y}$ for vertical direction). Similarly, the *i*-exposure spectrum deviation can be defined as ${{\rm{\Lambda }}}_{S}(i)=\int {\sigma }_{{S}_{i}}(E)dE/[i\times \int \bar{S}(E)dE]$, where ${\sigma }_{{S}_{i}}$ is the standard deviation of *n* groups of *i*-exposure spectra, and $\bar{S}(E)$ is the average spectrum of all the 80 shots. On Fig. 4c, Λ_*ph*_, ${{\rm{\Lambda }}}_{{\theta }_{x}}$ and ${{\rm{\Lambda }}}_{{\theta }_{y}}$ are plotted against the exposure number *i*, and these plots clearly show that the fluctuations of photon number and pointing decrease significantly with the increase of the exposure number *i*. For 15 exposures, the deviations of photon number and pointing have been reduced down to below 5%. On Fig. 4d, Λ_*S*_ is plotted against the exposure number *i*, and it is evident that the fluctuation of the spectrum has been significantly reduced from 40% level down to 10% level.

A statistical analysis with the parameter estimation method^36^ can be adopted to understand the above observed trend quantitatively. For a normal distribution, the minimal exposure number *m* required for a given allowed relative error Δ_*ph*_ of photon number can be estimated as^36^ $m={z}_{\alpha /2}^{2}RS{D}_{ph}^{2}/{{\rm{\Delta }}}_{ph}^{2}$. Here the relative standard deviation of the photon number (*RSD*_*ph*_) can be directly calculated as 16% from the measured data in Fig. 3b, and *z*_α/2_ is the critical value determined by the confidence level 1 − α. For one-σ confidence level (68%), *z*_α/2 _= 1 and Δ_*ph*_ = Λ_*ph*_. In Fig. 4c, *m* vs. Δ_*ph*_ for a confidence level of 68% is plotted (purple dashed line) for a direct comparison with the experimentally measured Λ_*ph*_ (red solid line), and one can see that the overall agreement is reasonably good.

### High quality phase-contrast imaging with 5-*µ*m resolution

**Figure 5:**
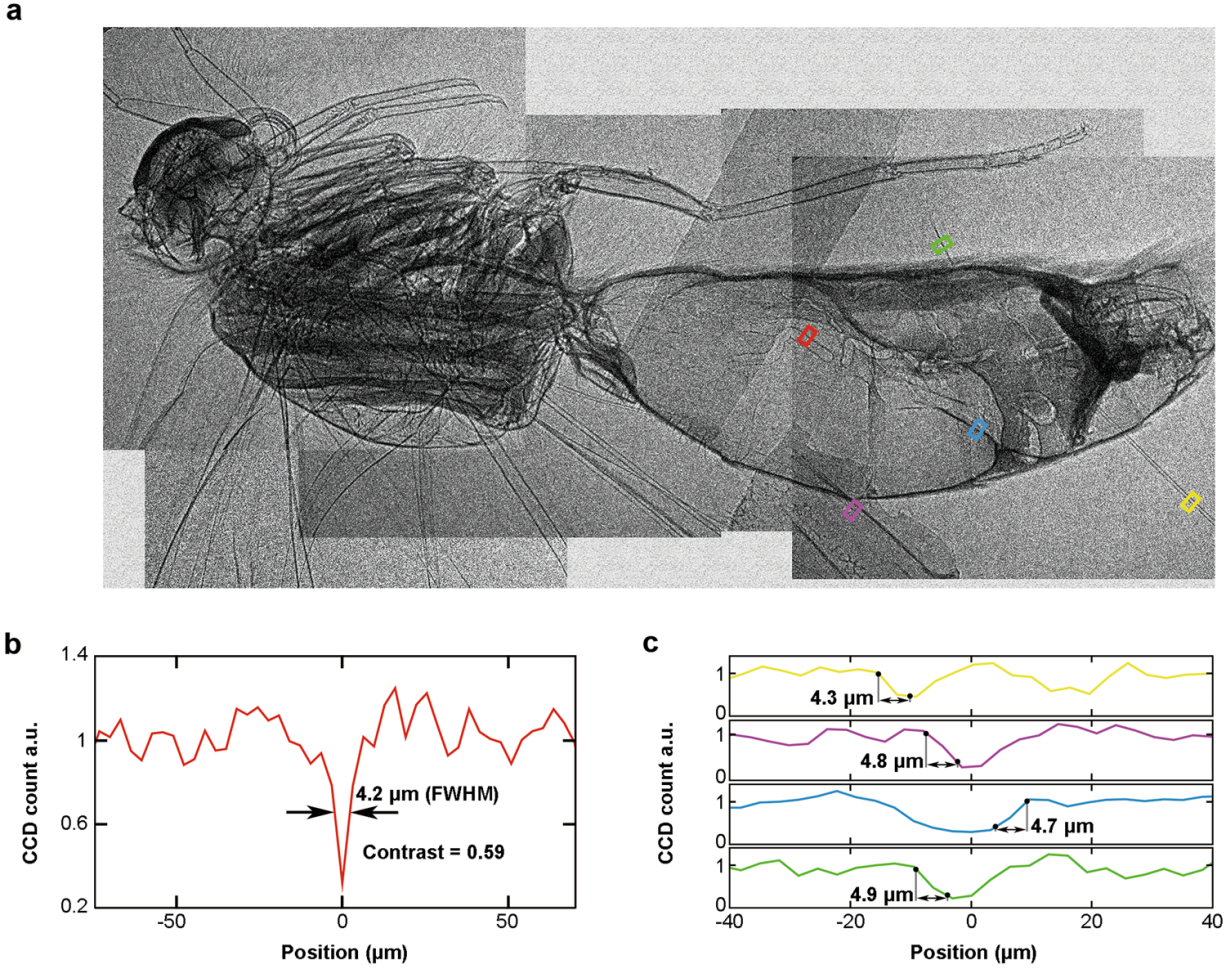
(**a**) X-ray phase contrast image of a 1-cm long butterfly under ionization injection condition, which is composed of 5 sub-images taken separately, and each sub-image was obtained by 10 exposures. (**b**) Lineout of the red square region in (**a**). A narrow dip of the gastrointestinal wall with a 4.2-*µ*m width (FWHM) can be clearly identified and measured with a contrast of 0.59. (**c**) Lineouts of the yellow, magenta, blue and green square regions in (**a**). The sharp edges with widths of ∼5 *µ*m (10% to 90% *H* − *L*) were measured.

To test the imaging capability of the betatron source obtained above, we have systematically carried out multiple-exposure phase-contrast imaging experiments for biological specimens (1-cm fish for 20 exposures, 1-cm butterfly for 10 exposures), and found that high-quality images can be obtained, as shown in Figs 1a and 5a. To evaluate the experimentally achieved imaging resolution, we take five lineouts on different portions of the same sub-image of the butterfly (red, yellow, magenta, blue and green squares in Fig. 5a), and plot them on Fig. 5b,c. From Fig. 5b, a narrow dip with a 4.2-*µ*m width (FWHM) can be clearly identified and measured with a contrast *C* = (*H* − *L*)/(*H* + *L*) = 0.59, where *H* and *L* are the peak and the valley of the signal. Figure 5c, sharp edges (10% to 90% *H* − *L*) of ~5-*µ*m width were measured, suggesting that 5-*µ*m resolution has been globally achieved in a same image.

## Discussion

**Figure 6:**
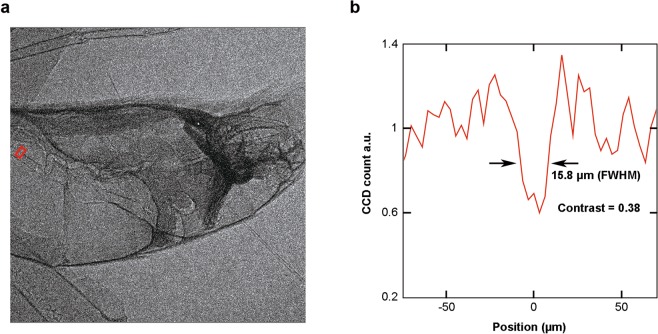
(**a**) 10-exposure X-ray phase contrast image with the self-injection based betatron source. (**b**) Lineout of the red square region in (**a**). A dip of the gastrointestinal wall with a 15.8-*µ*m width (FWHM) can be measured with a contrast of 0.38.

Compared with previous phase-contrast imaging results using betatron sources^23–25^, the resolution obtained in this work (5 *µ*m) is better than the previously reported resolutions in single-exposure imaging experiments (6–70 *µ*m). This is also in contrast to the previous observation that multiple-exposure images have much reduced contrast and resolution than that of single-exposure images using self-injection LWFA. To confirm the image quality improvement of multiple-exposure PCI with the ionization injection based betatron source, we have also carried out multiple-exposure phase-contrast imaging with a self-injection based betatron source under similar physical conditions (plasma electron density of 1.0 × 10^19^ cm^−3^ using pure helium gas, and with the same laser power). In Fig. 6a, a 10-exposure phase-contrast image of a portion of the butterfly (the same part as shown in one sub-image of Fig. 5a) with the self-injection based betatron source is plotted. Comparing with Fig. 5a, it is evident that some detailed features clearly visible in Fig. 5a disappear. In Fig. 6b, a lineout of the gastrointestinal wall of the butterfly (red square in Fig. 6a, the same part as the red square in Fig. 5a) is plotted, suggesting a significant degradation of the image resolution (15.8 *µ*m) and contrast (0.38) caused by the larger fluctuations of the X-ray source parameters.

**Figure 7:**
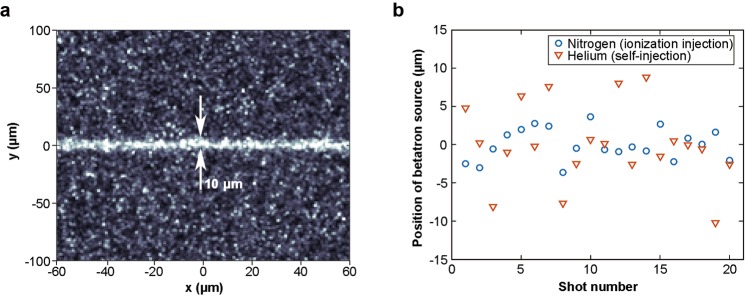
(**a**) A typical X-ray transmission image of the 10-*µ*m slit with the ionization injection based betatron source. (**b**) Positions of betatron source in ionization injection (blue circles) LWFA and self-injection (orange triangles) LWFA.

For the multiple-exposure imaging, the spatial resolution of the image is limited by the effective average source size that can de deduced from the shot-to-shot position fluctuation of the X-ray source. As a comparison, we measured the shot-to-shot position fluctuation of the betatron sources for ionization injection and self-injection respectively by imaging a 10-*µ*m slit. A typical X-ray image of the slit in ionization injection is plotted in Fig. 7a, and the measured positions of the X-ray source are shown in Fig. 7b. From Fig. 7b, it can be seen that the ionization-injection based betatron source has a much smaller shot-to-shot position fluctuation (2.0 *µ*m r.m.s.) compared to the self-injection based source (5.1 *µ*m r.m.s.), suggesting that ionization injection should be capable of providing higher spatial resolution for phase-contrast images.

Since betatron radiation is emitted continuously during the electron beam being accelerated, the emission length of the betatron radiation is another crucial factor for the degradation of image resolution. To estimate the effect of the emission length on image resolution, we used a technique of transverse laser machining^37,38^ (see Methods) to make a tomography for the emission region. The emission length was measured to be ∼200 *µ*m on the same experimental conditions, thus the maximum equivalent transverse source size caused by the combinations of the emission length (∼200 *µ*m) and the acceptance half-angle of CCD (∼10 mrad) was estimated to be ∼2 *µ*m.

The multiple-exposure phase-contrast images presented in this paper were accumulated without compensating the pointing jitters of individual images. Therefore, the maximum effective average source size can be calculated to be ∼ 5 *µ*m (FWHM) by convolving the deviation of source position with the equivalent transverse source size of single shot, which can potentially provide imaging with a 5-*µ*m resolution.

In the experiment, a clear PCI image was obtained within 1 minute at a repetition rate of ∼0.3 Hz (30 s for the butterfly, 60 s for the fish), with a photon number of 1.1 × 10^7^ per shot. This data acquisition time can be significantly reduced in future experiments by a factor of 10–300, through single shot photon number enhancement (10 times enhancement under similar conditions has been recently demonstrated^39^) and repetition rate increasing to 1 Hz^40^ to 10 Hz. For comparison with a typical microfocus X-ray tube of constant flux (source size 5 *µ*m, photon flux ∼2 × 10^4^ mrad^−2^ s^−1^)^41^, a continuous exposure time of 1 minute is needed to get an image with similar quality. Therefore, PCI based on betatron sources can potentially achieve 10–300 times faster speed than the typical microfocus X-ray tubes.

In summary, we demonstrate that a highly stable betatron source, with an effective average source size of ∼5 *µ*m, photon number and pointing jitter better than 5% and spectral fluctuation less than 10%, can be obtained by combining ionization injection in pure nitrogen gas with multiple exposures using a 30–40-TW laser. Using this source, high quality phase-contrast images of biological specimens with a 5-*µ*m resolution are obtained for the first time. This work paves the way for the application of high resolution phase-contrast imaging in biology and material science with stable betatron sources using small scale high repetition-rate lasers.

## Methods

### Phase-contrast imaging

To obtain the optimal contrast, the source to object distance *R*_1_ and the object to detector distance *R*_2_ need to be properly chosen to satisfy $\lambda \frac{{R}_{1}{R}_{2}}{{R}_{1}+{R}_{2}}({f}_{x}^{2}+{f}_{y}^{2})=\frac{1}{2}$ (the term of contrast determined by the phase in the transfer function of an in-line PCI^42,43^), where *λ* is the wavelength of the X-rays, and *f*_*x*_ and *f*_*y*_ are the maximal spatial frequency determined by the CCD pixel sizes. In our experiment, the CCD pixel size *δ* is 13 *µ*m, and the distance from the source to the detector (*R*_1_ + *R*_2_) is predetermined by the experimental setup as 0.96 m. Under these conditions, only one group of solution (*R*_1_ = 0.23 m, *R*_2_ = 0.73 m) exists for achieving optimal contrast. This group of parameters also determine the magnification factor *M* = (*R*_1_ + *R*_2_)/*R*_1_ = 4.2 and the limiting resolution of the imaging system (3.1 *µ*m, *δ*/*M*).

### Particle-in-cell simulation

Two-dimensional numerical simulations were carried out with the particle-in-cell code OSIRIS^32^, where a linearly polarized laser pulse (FWHM duration 36 fs) with a bi-Gaussian spatial profile is focused into a pure nitrogen gas target with a focal spot size of 16 *µ*m (FWHM) and a normalized vector potential *a*_0_ = 2.17. The density profile of the nitrogen atom increases linearly from zero to *n*_*N*_ = 2.0 × 10^18^ cm^−3^ in the front edge (0.5 mm), then keeps constant for 3 mm, and finally drops linearly to zero in the back edge (0.5 mm). The simulation window has a dimension of 127 × 64 *µ*m^2^ with 5000 × 2000 cells in the *x* and *z* directions, respectively. The N^(1–5)+^ and N^6+,7+^ ions are represented by 4 particles per cell, respectively.

### Tomography of the X-ray emission process

**Figure 8:**
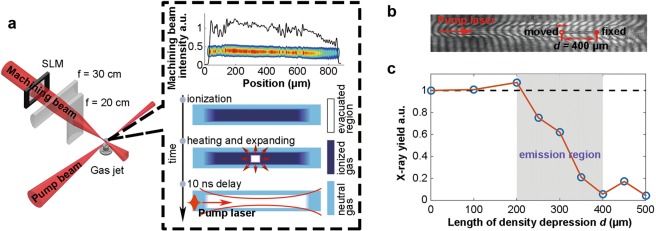
(**a**) Schematic of experimental layout for the tomography of X-ray emission. The insets within the black-dashed square show a typical intensity distribution and pattern of a shaped machining beam focus, indicating the principle for the creation of a plasma density depression in a gas jet. (**b**) A typically detected interferogram of plasma with a 400-*µ*m density depression. The length of density depression was scanned by moving the starting point of the density depression (red hollow circle) and fixing the end point (red dot). (**c**) Dependence of the measured X-ray yield on the length of density depression. Each point was measured by accumulating 10 shots. The grey region represents the emission region of betatron X-rays. The black dashed line represents X-ray yield without density depression.

To probe the emission region of betatron radiation, a tunable plasma density depression was created by another perpendicularly propagating machining beam^37,38^ (energy of 20–50 mJ and duration of 50–100 fs) to locally disrupt the X-ray emission. Figure 8a shows the schematic of the experimental layout for the tomography of X-ray emission and principle for the fabrication of a plasma density depression in the gas jet. The machining beam was firstly shaped by a programmable spatial light modulator (SLM), then focused vertically by a cylindrical lens (focal length f = 30 cm) to 20-*µ*m FWHM width and imaged horizontally by another cylindrical lens (f = 20 cm) from the location of SLM onto the path of pump beam with a demagnification of 10. When the shaped high-intensity machining beam was focused onto a neutral gas jet, plasma was formed and heated. After several nanoseconds (10 ns in our experiment), the plasma density of the machined region was reduced due to the expansion of the hot plasma, leading to a gas density depressed region for the subsequent pump laser. The boundary width of the depression can reach down to ∼10 *µ*m^37^. The length and position of the density depression can be conveniently adjusted by the SLM and the focusing cylindrical lens, respectively.

By scanning the length *d* of density depression along the path of pump beam, a tomography of the X-ray emission process can be obtained. Figure 8b gives a typically detected interferogram of plasma with a 400-*µ*m density depression. Figure 8c shows the dependence of the measured X-ray yield on the length of density depression. The emission length can be estimated to be ∼200 *µ*m according to the evolution of X-ray yield.
